# Age and duration of hypertension are associated with carotid artery tortuosity

**DOI:** 10.3389/fneur.2024.1307984

**Published:** 2024-03-11

**Authors:** Huan Huang, Jian-Jiong Fu, Pi-Guang Yao, Meng-Yao Wang, Xue Wang, An-Na Guo, Wei Li, Shao-Huai Chen, Dan-Dong Li

**Affiliations:** ^1^Department of Radiology and the Second Affiliated Hospital and Yuying Children's Hospital of Wenzhou Medical University, Wenzhou, Zhejiang, China; ^2^Department of Neurology, Zhuji Affiliated Hospital of Shaoxing University, Shaoxing, Zhejiang, China; ^3^Department of Neurology, Wenzhou Dongtou People’s Hospital, Wenzhou, Zhejiang, China; ^4^The First School of Medicine, Wenzhou Medical University, Wenzhou, Zhejiang, China; ^5^Department of Neurosurgery, The Second Affiliated Hospital and Yuying Children's Hospital of Wenzhou Medical University, Wenzhou, Zhejiang, China

**Keywords:** carotid artery tortuosity, computed tomography angiography, hypertension, aging, dolichoarteriopathy

## Abstract

**Objective:**

Tortuosity of the carotid artery is a common angiographic finding that may impact blood flow and neuronal function. However, information on its prevalence and risk factors remains limited. In this study, we determined to explore the factors affecting carotid artery tortuosity.

**Methods:**

The head and neck computed tomography angiography (CTA) imaging and cerebral angiography data performed at the Second Affiliated Hospital of Wenzhou Medical University between January 2019 and September 2021 were collected, and a total of 356 cases were enrolled in the study after screening. Carotid artery tortuosity refers to the angle between the two adjacent segments of the carotid artery, from the opening of the aortic arch on either side to the external orifice of the carotid canal, being less than 150°. A retrospective analysis was performed to compare the general information, laboratory indicators, personal history, and medical history between the two groups. The χ^2^ test, *t*-test, and Mann–Whitney *U*-test were performed to compare the parameters between the two groups. If there were significant differences between the groups, multivariate logistic regression was performed to analyze the factors affecting carotid artery tortuosity.

**Results:**

A total of 222 of the 356 cases were determined to have carotid artery tortuosity, accounting for 63.6%. There were statistically significant differences in age, body mass index (BMI), duration of diabetes and hypertension, levels of low-density lipoprotein cholesterol (LDL-C), diastolic blood pressure, history of ischemic and hemorrhagic stroke, and the usage of antihypertensive drugs between the two groups. Multivariate logistic regression analysis of the above factors showed that age (OR = 5.063, 95% CI 2.963–10.26, *p* < 0.001) and duration of hypertension (OR = 2.356, 95% CI 1.353–8.625, *p* = 0.021) were associated with a higher incidence of carotid artery tortuosity. Compared to patients who did not consume antihypertensive drugs, the incidence of carotid artery tortuosity was significantly less (OR = 0.094, 95% CI 0.002–0.713, *p* = 0.019) in those consuming antihypertensive drugs.

**Conclusion:**

Carotid artery tortuosity is a relatively common carotid artery disease. The incidence of carotid artery tortuosity may increase with age and the duration of hypertension. The consumption of antihypertensive drugs may reduce the incidence of carotid artery tortuosity.

## 1 Introduction

**Figure 1:**
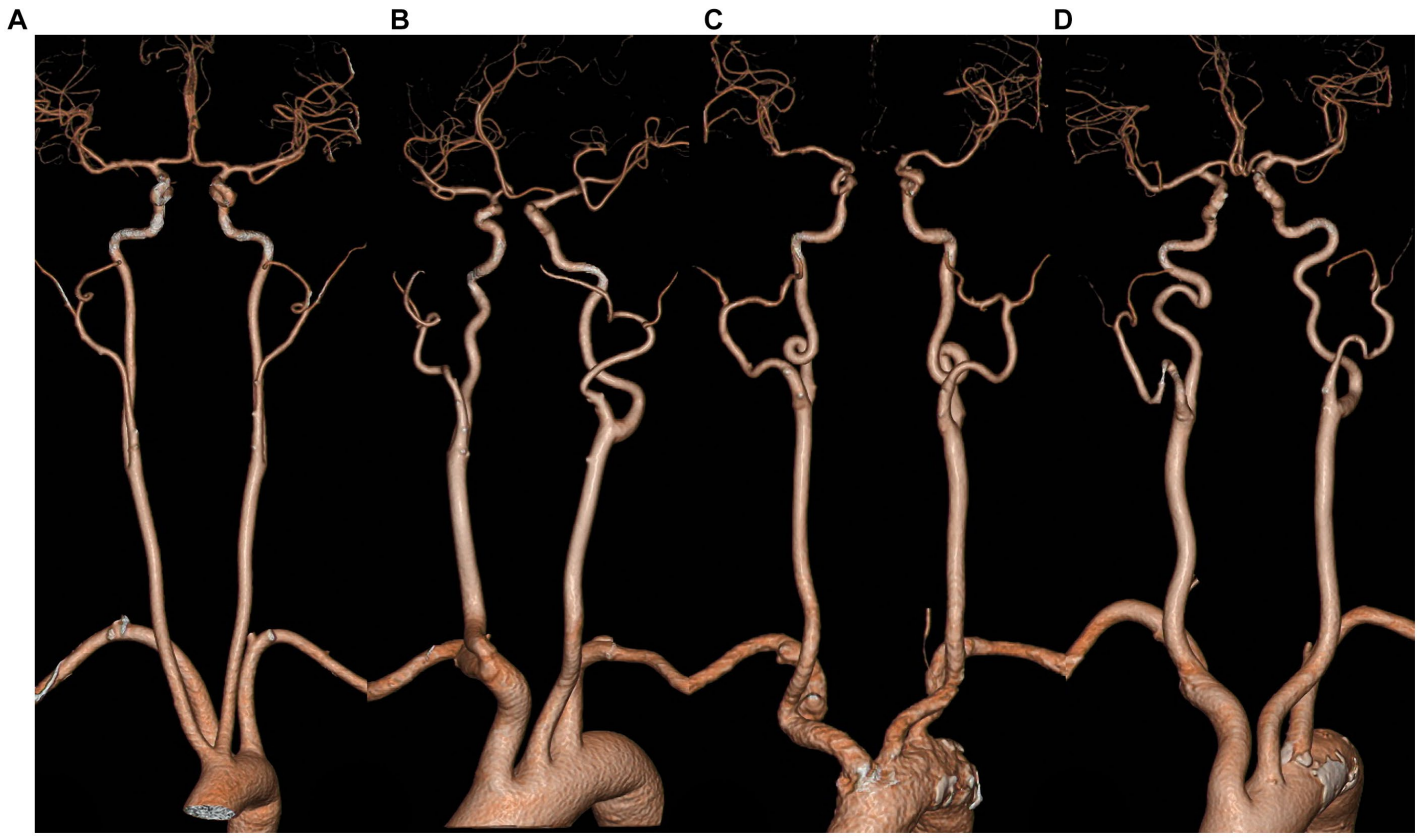
Classification of the morphology of carotid artery tortuosity. **(A)** normal; **(B)** tortuosity; **(C)** coiling; **(D)** kinking.

Carotid artery tortuosity, also known as dolichoarteriopathy, is a common clinical cerebrovascular morphological abnormality that was first reported by otolaryngologist as a special case. He accidentally injured the twisted and displaced carotid artery during a throat surgery, which caused a massive hemorrhage (1). Even now, the twisted and displaced carotid artery is still a major concern prior to neck surgery (2). In 1965, Weibel and Fields categorized carotid artery distortion into three types (1), namely tortuosity, where the carotid artery is “C”-, “U”-, or “S”-shaped; coiling, where the carotid artery is coiled into one or more arterial loops around an axis; and kinking, where the artery is reflexed into an acute angle or a “V” shape, as shown in Figure 1. With the development of imaging technology and the popularity of non-invasive cerebrovascular examinations, more and more patients with carotid artery tortuosity have been identified. It has been reported that the incidence rate of carotid artery tortuosity in elderly patients (over 60 years) is as high as 85% (3). In the past, carotid artery tortuosity was thought to be a benign lesion that did not lead to significant clinical symptoms, so there are few studies related to it.

It was reported that carotid tortuosity can reduce the blood supply to the brain by lowering blood pressure, and the degree of blood pressure loss was mainly correlated with the degree of carotid tortuosity. When the carotid artery angle was 60°, the blood flow could be reduced by more than 40%. When the angle is 30°, it can be reduced by more than 60%. The same trend was observed in the *in vitro* experiments and numerical simulation results. Since the human body has a self-regulatory mechanism that can offset part of the results of carotid tortuosity, the clinical observations are not quite consistent with the results of *in vitro* studies. However, when these autoregulatory mechanisms are weakened by some factors, such as hypertension, old age, and cardiovascular diseases, there will be a higher risk of stroke (4–7). Moreover, more and more studies have found that carotid artery tortuosity is associated with a variety of cerebrovascular diseases, including cerebral aneurysms and arterial dissection (8–12). Especially with the development of cerebrovascular intervention techniques, it has been found that carotid artery tortuosity complicates interventional treatment. The greater the twisted angle of the carotid artery, the more difficult it is for the catheter to pass, and it may even lead to surgical failure. Moreover, the incidence of complications, such as vasospasm and arterial dissection, during the surgery also increases significantly (13, 14). Therefore, carotid artery tortuosity has attracted the attention of clinicians. In this study, we focused on the factors affecting the occurrence of carotid artery tortuosity.

## 2 Data and methods

### 2.1 Clinical data

The computed tomography angiography (CTA) and cerebral angiography data from the Second Affiliated Hospital of Wenzhou Medical University uploaded between January 2019 and September 2022 were retrospectively screened and analyzed. All imaging data were independently interpreted by two physicians, and a third physician would cast the deciding vote in case of a conflicting result. Inclusion criteria were as follows: 1. The imaging data for head and neck blood vessels were complete. The images showed the carotid artery from the opening of the aortic arch to the cranial entry and the right carotid artery from the brachiocephalic trunk; 2. A complete medical and personal history was available; 3. All laboratory test results were available. The exclusion criteria were as follows: 1. blurred images; 2. occlusion of one or both carotid arteries or other lesions resulting in unclear carotid artery imaging. The collected clinical data included gender, age, blood pressure, blood sugar, blood lipids, history of stroke, body mass index (BMI), smoking index, and drinking index. Moreover, the presence of other cerebrovascular diseases, such as aneurysm, arterial dissection, and stenosis, was recorded.

### 2.2 Imaging protocol

The CTA scans were acquired using a 256-slice CT scanner (Philips Brilliance; Philips Medical Systems, Best, the Netherlands). All patients underwent an examination in the supine position with their heads immobilized by a head holder to minimize motion artifacts. Visualization of the carotid arteries extends from the inferior margin of the aortic arch to the superior margin of the cranial vault. The imaging parameters were as follows: pitch 0.606; section collimation 0.6 mm; reconstruction section thickness and interval 0.625 mm; matrix size 512 × 512; field of view 180–250 mm; tube voltage of 120 kV; and tube current of 150 mA. Intravascular contrast (iopromide) was injected at a volume of 65 mL, followed by a saline bolus injection of 40 mL, both administered at a flow rate of 6 mL per second. Multiplanar reconstruction was performed on a three-dimensional (3D) workstation (Philips Medical Systems).

### 2.3 Identification of the factors affecting carotid artery tortuosity

**Figure 2:**
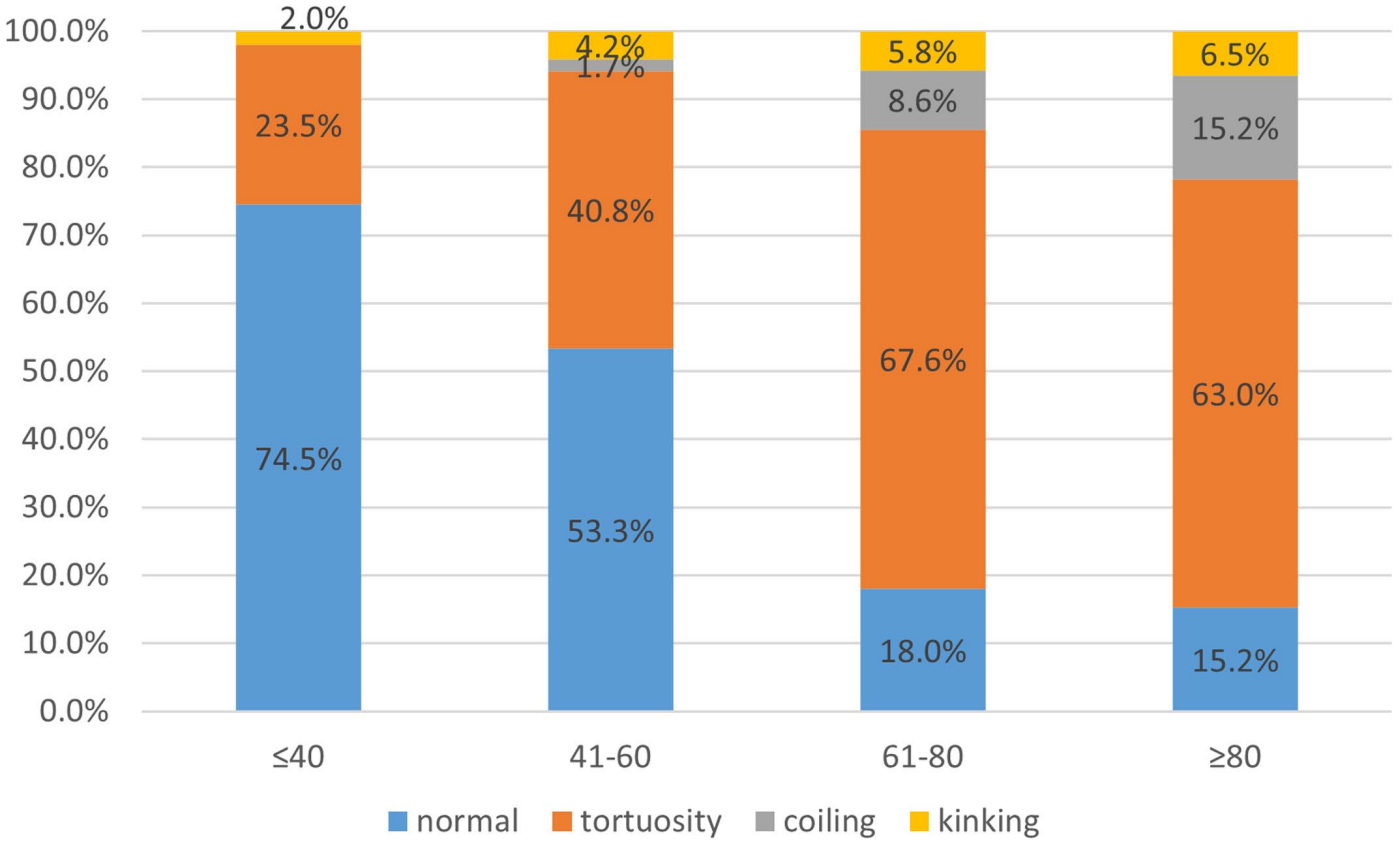
Percentages of different kinds of tortuosity in each age group.

Carotid artery tortuosity refers to the angle between two adjacent segments of the carotid artery from the carotid bifurcation to the external orifice of the carotid canal being less than 150°, and it is identified as tortuosity if either side coincides. The carotid artery angle was measured as shown in Figure 2, with an imaginary line following the carotid path at each coiled or curve on a suitable view.

Hypertension was defined as systolic blood pressure ≥ 140 mmHg, diastolic blood pressure ≥ 90 mmHg, and/or consumption of antihypertensive drugs. Diabetes was defined as a diagnosis made by a professional physician in the past, fasting blood glucose ≥7 mmol/L, and/or consumption of hypoglycemic drugs. Dyslipidemia was defined as triglycerides (TGs) ≥ 2.26 mmol/L, total cholesterol (TC) ≥ 6.22 mmol/L, low-density lipoprotein cholesterol (LDL-C) ≥ 4.14 mmol/L, high-density lipoprotein cholesterol (HDL-C) < 1.04 mmol/L, and/or consumption of lipid-regulating drugs. Hyperuricemia was defined as serum uric acid >420 μmol/L in men and > 360 μmol/L in women. The smoking index was calculated as the number of cigarettes smoked per day × the number of years of smoking (cigarette year). The drinking index was calculated as the grams of ethanol consumed per week × the number of years of drinking (grams per year). BMI was calculated as weight divided by height squared (kg/m^2^).

### 2.4 Statistical methods

SPSS 19.0 software was used for statistical analysis of the data. All measurement data were analyzed using the Kolmogorov–Smirnov test for normality, and the measurement data conforming to the normal distribution are expressed as mean ± SD. A *t*-test was performed for comparison between the groups. The non-parametric Mann–Whitney *U*-test was performed to compare the measurement data with a skewed distribution. The numerical data are expressed as the number of cases and percentages, and the groups were compared using the χ^2^ test. Multivariate logistic regression was performed to analyze the factors affecting the carotid artery tortuosity. A receiver operating characteristic curve was used to calculate the area under the curve and determine the optimal cutoff value for the prediction of carotid artery tortuosity. A *p*-value of <0.05 was considered statistically significant.

## 3 Results

### 3.1 General information

**Figure 3:**
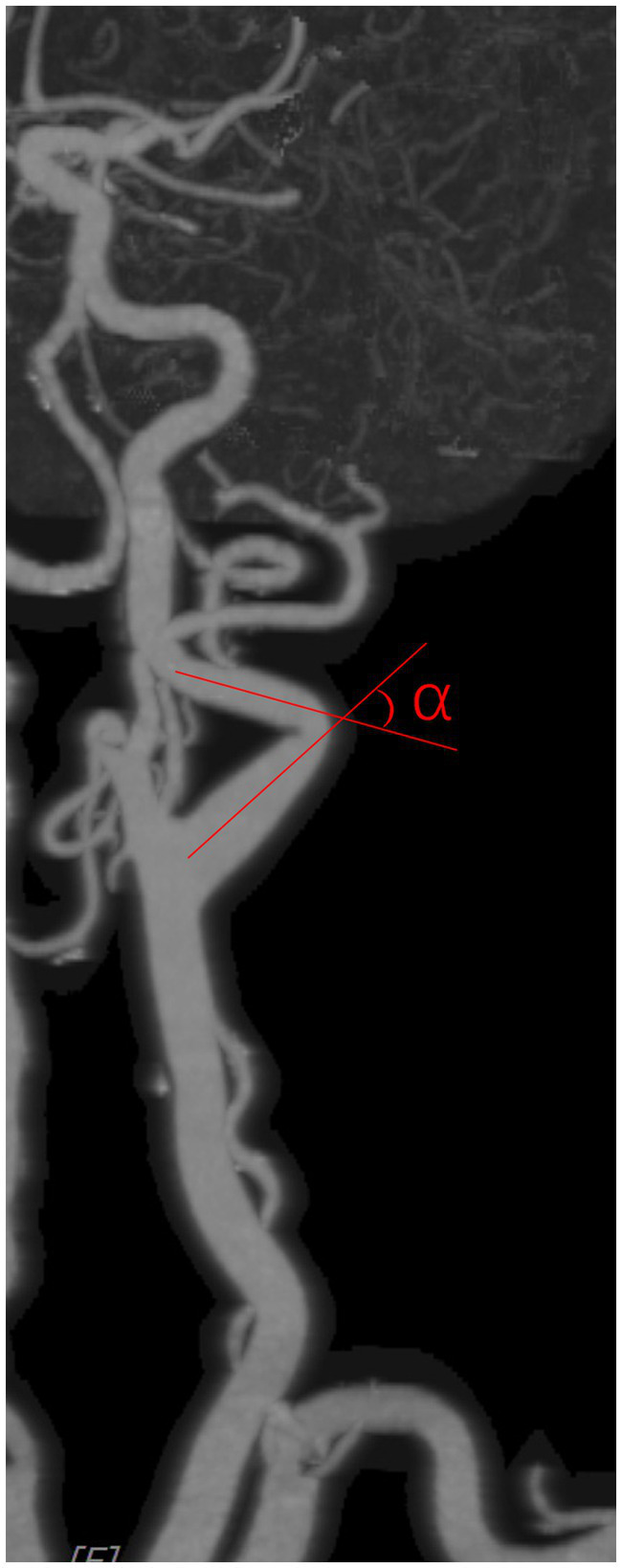
Carotid artery angle was measured as shown in the figure, with an imaginary line following the carotid path on a suitable view.

A total of 356 cases were included in the study, of which 222 were part of the carotid artery tortuosity group (62.4%) and 134 cases in the normal group (37.6%). There were 235 male subjects in total, accounting for 66.0%. There was no significant difference in gender between the two groups. The average age was 63.9 ± 12.4, with a minimum age of 14 years and a maximum age of 98 years in general. The proportion of patients with carotid artery tortuosity was 25.5% (13/51) in those ≤40 years old, 46.7% (56/120) in those 41–60 years old, 82.0% (114/139) in those 61–80 years old, and 84.8% (39/46) in those ≥80 years old, suggesting that age significantly affected carotid artery tortuosity (*p* < 0.001), as shown in Table 1; Figure 3. The BMI of patients in the carotid artery tortuosity group was significantly higher than that in the normal group (25.41 ± 4.36 vs. 23.18 ± 3.12, *p* = 0.043). There were no significant differences in height, smoking index, or drinking index between the two groups, as shown in Table 1.

**Table 1:** Baseline characteristics.

	ICA tortuosity (*n* = 222)	ICA normal (*n* = 134)	*p*
Sex (male, %)	140 (63.6%)	95 (43.2%)	0.131
Age (year)	67.25 ± 13.50	55.13 ± 14.11	<0.001
≤40	13	38	
41–60	56	64	
61–80	114	25	
≥81	39	7	
Height (cm)	166.8 ± 10.5	165.3 ± 12.7	0.872
BMI (kg/m^2^)	25.41 ± 4.36	23.18 ± 3.12	0.043
Smoking index (cigarette/ year)	326.71 ± 23.97	335.39 ± 35.29	0.105
Alcohol index (gram/ year)	918.63 ± 124.02	871.82 ± 90.34	0.635
Diabetes	83 (37.4%)	35 (26.1%)	0.029
FBG (mmol/L)	6.78 ± 1.14	6.20 ± 1.69	0.520
GHB (%)	5.12 ± 1.98	5.58 ± 2.23	0.413
Diabetes duration (year)	4.32 ± 2.63	3.36 ± 2.80	0.236
Hypertension	137 (61.7%)	64 (47.8%)	0.01
SBP (mmHg)	158.83 ± 28.63	157.47 ± 22.31	0.754
DBP (mmHg)	77.19 ± 9.20	70.62 ± 10.02	0.001
Hypertension duration (year)	8.26 ± 2.77	4.38 ± 2.50	<0.001
Dyslipidemia	94 (42.3%)	54 (40.3%)	0.705
TC (mmol/L)	4.69 ± 2.77	4.86 ± 1.93	0.059
TG (mmol/L)	1.52 ± 0.37	1.56 ± 0.62	0.698
HDL-C (mmol/L)	1.37 ± 0.70	1.42 ± 0.45	0.510
LDL-C (mmol/L)	3.00±0.76	2.42 ± 1.02	0.040
Dyslipidemia duration (year)	2.35±1.21	2.51 ± 1.53	0.713
Hyperuricemia	27 (12.2%)	25 (18.7%)	0.093
BUA(μmol/L)	321.62 ± 94.82	339.39 ± 75.42	0.352
Hyperuricemia duration (year)	0.52 ± 0.35	0.50 ± 0.21	0.821
History of stroke	145 (65.3%)	76 (56.7%)	0.105
Ischemic	129 (58.1%)	43 (32.8%)	0.001
Hemorrhagic	21 (9.46%)	34 (25.4%)	0.018
History of cardiovascular disease	21 (9.46%)	12 (8.90%)	0.874
Medication history	182 (82.0%)	102 (76.1%)	0.182
Antidiabetic	53 (23.9%)	36 (26.9%)	0.528
Antihypertensive	42 (18.9%)	46 (34.3%)	0.001
Antihyperlipidemic	62 (27.9%)	47 (35.1%)	0.156
Antiplatelet	82 (36.9%)	58 (43.3%)	0.235
Anticoagulant	18 (8.1%)	16 (11.9%)	0.233
Antihyperuricemic	8 (3.6%)	11 (8.2%)	0.061
Combined arterial diseases (aneurysm/dissection/malformation)	72 (32.4%)	53 (39.5%)	0.173
Carotid artery stenosis	138 (62.2%)	75 (56.0%)	0.248

BMI, body mass index; FBG, fasting blood glucose; GHB, glycosylated hemoglobin; SBP, systolic pressure; DBP, diastolic pressure; TC, total cholesterol; TG, triglyceride; HDL-C, high-density lipoprotein cholesterol; LDL-C, low-density lipoprotein cholesterol; BUA, blood uric acid.

### 3.2 Results of the univariate analysis

The number of patients with diabetes in the carotid artery tortuosity group was significantly greater than that in the normal group (*p* = 0.029), but there were no significant differences in fasting blood glucose, glycosylated hemoglobin, or the duration of diabetes between the two groups (all *p* > 0.05). The carotid artery tortuosity group (61.7%, 137/222) had more patients with hypertension compared to the normal group (47.8%, 64/134) (*p* = 0.01). Moreover, there were significant differences in diastolic blood pressure (*p* = 0.001) and course of hypertension (*p* < 0.001) between the two groups, but there was no difference in the systolic blood pressure (*p* > 0.05) between the two groups. There were no significant differences in the number of patients with TC, TG, and HDL-C concentrations, hyperlipidemia, and hyperlipidemia duration between the two groups (all *p* > 0.05); however, the LDL-C concentration in the carotid artery tortuosity group was significantly higher than that in the normal group (3.00 ± 0.76 vs. 2.42 ± 1.02, *p* = 0.040). There was no significant difference in the number of hyperuricemia patients, blood uric acid values, or hyperuricemia duration between the two groups (*p* > 0.05). The number of patients with a history of stroke was not statistically different between the two groups. The proportion of patients with ischemic stroke in the carotid artery tortuosity group (58.1%, 129/222) was significantly higher than that in the normal group (32.8%, 43/134) (*p* = 0.001), while the number of hemorrhagic stroke patients in the normal group was higher (*p* = 0.018). There was no significant difference in the number of patients with cardiovascular disease or medication history between the two groups (all *p* > 0.05). The proportion of patients consuming antihypertensive drugs in the normal group (63.4%, 85/134) was higher than that in the carotid artery tortuosity group (23.4%, 52/222) (*p* = 0.015). There was no significant difference in the number of patients with other medication histories between the two groups. There was no significant difference in the incidence of combined arterial diseases, such as cerebral aneurysms and combined carotid artery stenosis, between the two groups (all *p* > 0.05). The above data are shown in Table 2.

**Table 2:** Baseline characteristics.

Risk factors	*B*	OR	95%CI	*p*
Age	1.622	5.063	2.963–10.026	<0.001
BMI	0.038	1.039	0.854–3.951	0.134
Diabetes	0.209	1.232	0.642–3.415	0.068
Hypertension	0.648	1.912	0.902–5.247	0.054
DBP	0.325	1.384	0.710–5.562	0.113
Hypertension duration	0.857	2.356	1.353–8.625	0.021
LDL-C	1.428	4.170	0.723–10.427	0.763
History of ischemic stroke	0.951	2.588	0.895–4.251	0.406
History of hemorrhagic stroke	−0.441	0.643	0.038–1.694	0.528
Antihypertensive drugs	−2.365	0.094	0.002–0.713	0.019

BMI, body mass index; DBP, diastolic pressure; LDL-C, low-density lipoprotein cholesterol.

### 3.3 Multivariate logistic regression analysis of The factors affecting carotid artery tortuosity

Multivariate logistic regression analysis was performed by considering carotid artery tortuosity as the dependent variable and factors showing a significant association in the univariate analysis, such as age, BMI, diabetes, hypertension, diastolic blood pressure, duration of hypertension, LDL-C, history of ischemic stroke, history of hemorrhagic stroke, and the use of antihypertensive drugs, as independent variables. The results showed that age (OR = 5.063, 95% CI 2.963–10.26, *p* < 0.001) and duration of hypertension (OR = 2.356, 95% CI 1.353–8.625, *p* = 0.021) were independent risk factors for carotid artery tortuosity, while the use of antihypertensive drugs (OR = 0.094, 95% CI 0.002–0.713, *p* = 0.019) was an independent protective factor against carotid artery tortuosity, as shown in Table 2.

**Figure 4:**
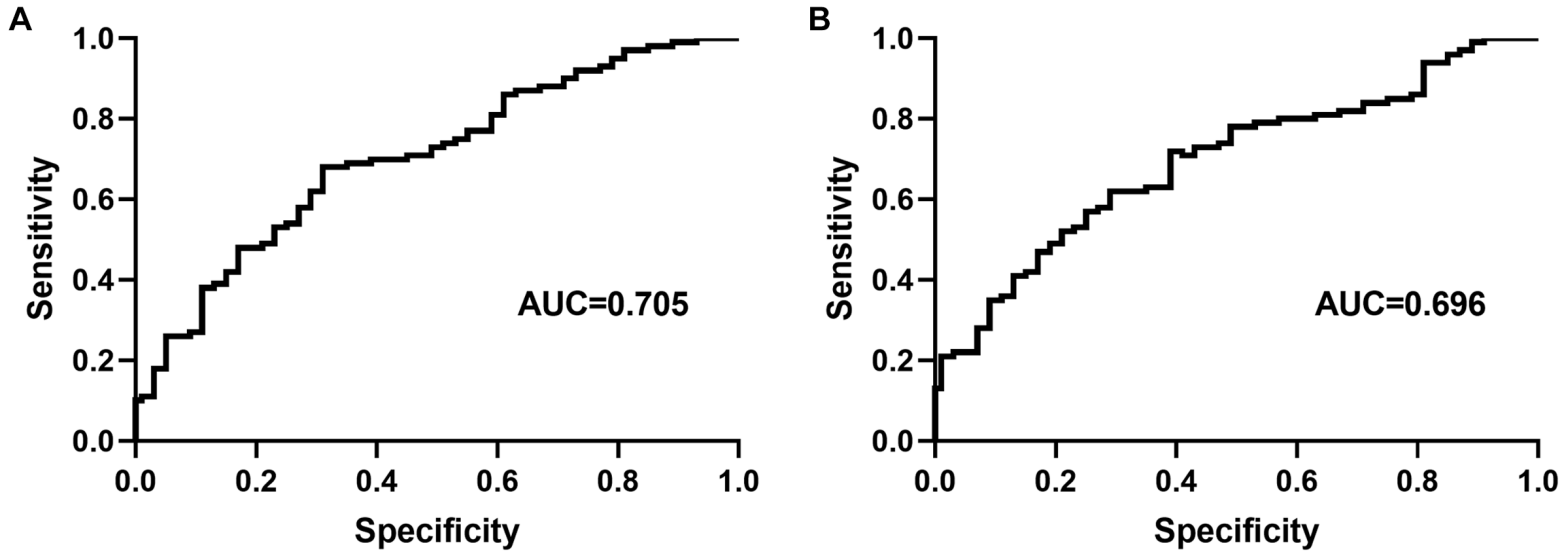
Receiver operating characteristic curve for age **(A)** and duration of hypertension **(B)** for predicting carotid artery tortuosity. AUC = area under the curve.

At the optimal cutoff age value of 78.6, where the values of sensitivity and specificity were considered maximal, the sensitivity and specificity were 67 and 73%, respectively. When the duration of hypertension values was at the optimal cutoff of 10.8, the sensitivity and specificity were 45 and 82%, respectively (Figure 4).

## 4 Discussion

Mild carotid artery tortuosity does not cause clinical symptoms and has a slight impact on cerebral blood flow under normal circumstances, but some severely distorted, kinked, or coiled arteries affect hemodynamics, and the pressure of blood flow decreases significantly after passing through these vessels, thus affecting cerebral perfusion (4). Moreover, turbulence occurs when the blood flows through severely distorted blood vessels, which leads to thrombosis and subsequent cerebral infarction (5). In addition, carotid artery tortuosity has more practical significance for neurointerventional doctors. When intravascular interventional therapy encounters a kinked or coiled artery, the treatment difficulty, radiation exposure time, complications, and even surgical failure rate increase significantly (14, 15). Moreover, during mechanical thrombectomy in acute cerebral infarction, patients with carotid artery tortuosity require a higher surgical time and have a worse prognosis (15, 16). Therefore, with the development of cerebrovascular interventional therapy, people have to pay attention to this phenomenon.

The pathogenesis of carotid artery tortuosity is still unclear. It is generally believed to be related to both congenital and acquired factors. Studies have shown that carotid artery tortuosity is related to certain congenital diseases, such as Marfan syndrome and Ehlers–Danlos syndrome (17, 18). These basic diseases lead to structural changes or the loss of components in the vascular wall and vascular remodeling. Other studies have shown that the incidence of coiling and kinking is as high as 31% in children younger than 15 years old (19), which also suggests the role of congenital factors in carotid artery tortuosity. Moreover, the incidences of cerebral aneurysm, arterial dissection, aortic aneurysm, and vasodilation in other parts of the body in patients with carotid artery tortuosity were significantly higher than those patients without carotid artery tortuosity. Therefore, researchers believe that carotid artery tortuosity is a systemic vascular disease (18). In this study, we explored the effect of commonly acquired factors on carotid artery tortuosity.

In previous studies, the incidence rate of carotid artery tortuosity ranged from 4 to 87% (3, 12, 20). The incidence rate in this study was 63.6%, and such a large difference in the incidence rates across different studies may be due to the following reasons. First, the criteria for determining carotid artery tortuosity are different. There is no unified diagnostic standard. Currently, the standard proposed by Metz in 1961 is widely used (21), which defines the curvature of carotid artery tortuosity as <90° and classifies them based on the angle: Type I is 60–90°, Type II is 30–60°, and Type III is <30°. A few studies define the curvature of carotid artery tortuosity as <150° (22), while others define it as <165° (23). According to clinical practice, when the angle between two adjacent arteries is less than 150°, the passing of the catheter may be affected. Therefore, our criterion in the current study was set at <150°. Second, selection bias cannot be ignored. Similar to most previous studies, most of the enrolled cases were patients with cerebrovascular diseases, and these patients usually have several other diseases associated with age, all of which can lead to an increase in the incidence rate. In addition, the small sample size in the current study is also an important reason for the inconsistent results. Therefore, further large-scale epidemiological investigations are needed to understand the true incidence rate in the population.

We found that age and the duration of hypertension are independent risk factors for carotid artery tortuosity, while the use of antihypertensive drugs is a protective factor against carotid artery tortuosity. In addition to age, the duration of hypertension and antihypertensive drugs is related to blood pressure. Based on current research, although the acquired risk factors related to carotid artery tortuosity vary greatly among studies, age and hypertension are currently accepted by the majority of studies (3, 20, 24, 25). With age, the elastic layer of blood vessels degenerates and breaks, and their integrity is destroyed. Hypertension increases vascular resistance and sympathetic excitability, thereby affecting the proliferation of vascular smooth muscle cells. With the increase in the duration of hypertension, this effect is more significant. These two factors jointly affect vascular remodeling. The use of hypertensive drugs not only weakens the effects of hypertension on the vascular wall but also helps protect the blood vessels.

In conclusion, in the current study, it was found that age and the duration of hypertension are two risk factors for carotid artery tortuosity, and the use of antihypertensive drugs can reduce the incidence of carotid artery tortuosity in patients with hypertension. It is the first time to report the relationship between the duration of hypertension and carotid artery tortuosity. The present results highlight the long-term impact of one or several risk factors as the primary cause of carotid artery morphological changes.

## Data availability statement

The original contributions presented in the study are included in the article/supplementary material, further inquiries can be directed to the corresponding author.

## Ethics statement

The studies involving humans were approved by the Ethical Committee of the Second Affiliated Hospital of Wenzhou Medical University. The studies were conducted in accordance with the local legislation and institutional requirements. Written informed consent for participation was not required from the participants or the participants’ legal guardians/next of kin in accordance with the national legislation and institutional requirements.

## Author contributions

HH: Funding acquisition, Data curation, Formal analysis, Writing – original draft. J-JF: Conceptualization, Data curation, Formal analysis, Project administration, Writing – review & editing. P-GY: Data curation, Formal analysis, Investigation, Software, Writing – review & editing. M-YW: Data curation, Formal analysis, Writing – original draft. XW: Data curation, Formal analysis, Investigation, Writing – review & editing. A-NG: Data curation, Formal analysis, Writing – review & editing. WL: Data curation, Formal analysis, Software, Writing – review & editing. S-HC: Data curation, Investigation, Writing – review & editing. D-DL: Funding acquisition, Resources, Supervision, Writing – review & editing.
